# Characterization and ACE Inhibitory Activity of Fermented Milk with Probiotic *Lactobacillus plantarum* K25 as Analyzed by GC-MS-Based Metabolomics Approach

**DOI:** 10.4014/jmb.1911.11007

**Published:** 2020-03-27

**Authors:** Zhang Min, Jiang Yunyun, Cai Miao, Yang Zhennai

**Affiliations:** 1 Beijing Advanced Innovation Center for Food Nutrition and Human Health, Beijing Engineering and Technology Research Center of Food Additives, Beijing Technology and Business University, Beijing 00048, P.R. China; 2 Mengniu Dairy (Beijing) Co., Ltd., Beijing, P.R. China

**Keywords:** *L. plantarum* K25, fermented milk, metabolites, ACE inhibition

## Abstract

Addition of probiotics to yogurt with desired health benefits is gaining increasing attention. To further understand the effect of probiotic *Lactobacillus plantarum* on the quality and function of fermented milk, probiotic fermented milk (PFM) made with probiotic *L. plantarum* K25 and yogurt starter (*L. delbrueckii* ssp. *bulgaricus* and *Streptococcus thermophilus*) was compared with the control fermented milk (FM) made with only the yogurt starter. The probiotic strain was shown to survive well with a viable count of 7.1 ± 0.1 log CFU/g in the PFM sample after 21 days of storage at 4°C. The strain was shown to promote formation of volatiles such as acetoin and 2,3-butanediol with milk fragrance, and it did not cause post-acidification during refrigerated storage. Metabolomics analysis by GC-MS datasets coupled with multivariate statistical analysis showed that addition of *L. plantarum* K25 increased formation of over 20 metabolites detected in fermented milk, among which γ-aminobutyric acid was the most prominent. Together with several other metabolites with relatively high levels in fermented milk such as glyceric acid, malic acid, succinic acid, glycine, alanine, ribose, and 1,3-dihydroxyacetone, they might play important roles in the probiotic function of *L. plantarum* K25. Further assay of the bioactivity of the PFM sample showed significant (*p* < 0.05) increase of ACE inhibitory activity from 22.3% at day 1 to 49.3% at day 21 of the refrigerated storage. Therefore, probiotic *L. plantarum* K25 could be explored for potential application in functional dairy products.

## Introduction

There is an increasing interest in developing probiotic foods with different functionalities, such as regulating intestinal flora, lowering cholesterol levels, regulating blood glucose level, etc. [1]. Yogurt is a fermented dairy product consumed worldwide with high nutritional value and well-established health benefits, especially when reinforced with probiotic bacteria [2-5]. Addition of probiotic bifidobacteria and lactobacilli to yogurt has become a common practice nowadays in yogurt manufacturing, but there are concerns about maintaining probiotic viability considering unfavorable factors such as various mechanical stresses, low pH, low temperature, oxygen, etc., during yogurt processing and storage [6]. To deliver the health benefits to the consumers, it is of utmost importance to maintain viable probiotic cells during cold (about 6°C) storage with viable counts ranging from 10^6^ to 10^9^ CFU/ml in a probiotic product till consumption [7].

Metabolomics deals with simultaneous determination and quantitative analysis of intracellular metabolites that are produced and modified by the metabolism of living organisms (*i.e.* microbes). These metabolic compounds with low molecular mass (less than 1,500 Da) are not genetically encoded, including peptides, amino acids, nucleic acids, carbohydrates, organic acids, vitamins, polyphenols, alkaloids and minerals [5, 8]. Metabolomics have been successfully used in food science to evaluate the molecular fingerprints of fermented foods, such as soy products, cheeses, yogurt and wines, regarding food quality and maturity, as well as traceability and authenticity of the products [9]. For example, metabolomics analysis by high-resolution magic angle spinning-nuclear magnetic resonance (HRMAS-NMR) was performed with the serum and feces from individuals suffering from irritable bowel syndrome. The results revealed the effectiveness of treatment with fermented milk containing probiotic *Lactobacillus* and *Bifidobacteria* strains, which normalized alterations in blood levels of glucose, tyrosine and lactate after 2 months of treatment [10, 11].

In our previous study, we isolated and identified from Chinese Tibetan kefir grains the probiotic *L. plantarum* K25 strain with ability to inhibit biofilm formation of pathogens, lower serum cholesterol level and regulate intestinal microbiota in a mice model [12-14]. The whole genome sequencing of *L. plantarum* K25 was completed and the functional genes relevant to its probiotic properties were also analyzed [15]. However, little is known about the formation and change of the metabolites, or the relevant effects on volatiles and bioactivities of fermented milk with *L. plantarum* K25. There were several reports on the fermented milk with inhibitory activities on angiotensin converting enzyme (ACE) that prevented the conversion of Angiotensin I to Angiotensin II, leading to reduced blood pressure [13, 14]. ACE-inhibitory (ACEi) activity was reported on milk fermented with LAB such as probiotic fermented milk by *Bifidobacterium bifidum* MF 20/5 [16], *Enterococcus durans*, *L. acidophilus* and *L. rhamnosus* [17]. However, the ACEi activity of fermented milk with *L. plantarum* was less studied. Several bioactive components from food sources were proved to be antihypertensive, such as peptides formed during fermentation/enzymatic hydrolysis [19], γ-amino butyric acid (GABA) in fermented milk [20], and polyphenols from tea and cocoa [21].

In this study, fermented milk added with probiotic *L. plantarum* K25 was studied with respect to the probiotic survivability, volatile formation analyzed by SPME (solid-phase microextraction)–GC/MS (gas chromatography/mass spectrometry), and changes of metabolites analyzed by GC-MS-based metabolomics method. The relevant metabolic pathways in *L. plantarum* K25 in fermented milk were proposed. Furthermore, the ACEi activity of the probiotic fermented milk and the activity changes during refrigerated storage were also evaluated. The present study should provide further understanding on the cellular metabolism related to probiotic function and effect on the ACEi activity of the probiotic fermented milk.

## Materials and Methods

### Bacterial Cultures

Probiotic *L. plantarum* K25 isolated and identified from Tibetan kefir grains was obtained from the Northeast Agricultural Research Center of China, Changchun, China. The strain was stored in 30% (v/v) glycerol at -80°C. It was inoculated into MRS (Man Rogasa Sharpe) (Difco, USA) broth and grown at 37°C for 12 h to obtain a cell count of log 8-9 CFU/ml. This cultured broth was then used for inoculation at 2% (w/v) in reconstituted skim milk (12%, w/v) to prepare the starter for making fermented milk. The yogurt starter of *L. delbrueckii* ssp. *bulgaricus* and *Streptococcus thermophilus* in powder form was obtained from DANISCO, Denmark.

### Preparation of Fermented Milk

Fermented milk was prepared by heat-treating reconstituted skimmed milk (12% w/v) at 95°C for 5 min, followed by cooling to 45°C, and aseptically inoculating with 0.002 g/L of the yogurt starter. The inoculated milk was divided into 2 equal portions, one without inoculation of *L. plantarum* K25 as the control fermented milk (FM), and another one further inoculated with 2% (w/v) of probiotic *L. plantarum* K25 to prepare probiotic fermented milk (PFM). The mixes were poured into polystyrene cups aseptically and incubated at 37°C. Decrease of pH was monitored every 1.5-3 h till the required pH value of 4.5 ± 0.5 was reached, and then the samples were cooled to 4°C to stop further acidification. The fermented milk samples were stored at 4°C for 21 days (the typical shelf life of commercial yogurts). All samples were taken on the 1st, 7th, 14th and 21st days for the chemical and microbiological analyses as described below.

### Enumeration of Viable Bacteria

Viable counts expressed as CFU (colony forming unit) per gram of the fermented milk samples were determined by plate counting on MRS agar at 37°C for 24 h. *L. plantarum* K25 was enumerated on *L. plantarum* selective medium under anaerobic incubation at 37°C for 72 h [22].

### Measurement of pH Change

Production of acid during storage of the fermented milk samples was expressed by measuring changes of pH (pH-250L, ISTEK, Korea) [23].

### SPME–GC/MS Profiling

Headspace volatiles of fermented milk were evaluated by SPME- GC/MS. Each fermented milk sample (30 ml) was loaded in triplicate into 20 ml autosampler vials with steel screw tops containing silicone septa faced in Teflon (Microliter Analytical, USA). An internal standard solution (2-methyl-3-heptanone in methanol, Sigma-Aldrich, USA) was added to each vial to control for analysis of variability with samples of fermented milk running only after >4 h at storage temperature [24].

Samples were injected using a CombiPal autosampler (CTC Analytics, Zwingen, Switzerland) attached to an Agilent 6890N gas chromatograph with 5973 inert MS detection (Agilent Technologies Inc., USA). Samples were maintained at 10°C before fiber exposure. Samples were equilibrated at 40°C for 25 min before 30 min fiber exposure of a 1 cm divinylbenzene/carboxen/polydimethylsiloxane (DVB/CAR/PDMS) fiber at 31 mm with 4 s pulsed agitation at 250 rpm. Fibers were injected for 5 min at a depth of 50 mm. The GC method was performed at an initial temperature of 40°C for 5 min with a ramp rate of 8°C/min to 250°C held for 5 min. The SPME fibers were introduced into the split/splitless injector at 250°C. An Rtx-5ms column (30 m length × 0.25 mm inner diameter × 0.25 μm film thickness; Restek, USA) was used for all analyses at a constant helium flow rate of 1 ml/min. Purge time was set at 1 min. The MS transfer line was maintained at 250°C with the Quad at 150°C and Source at 250°C.

### Derivatization

L-norleucine, N,O-bis(trimethylsilyl)trifluoroacetamide with 1% trimethylchlorosilane [BSTFA (1% TMCS)], methoxyamine hydrochloride, and anhydrous pyridine were purchased from Sigma-Aldrich. A 20 μl thawed sample and 80 μl of cold methanol including internal standard (5 μg/ml L-norleucine) were combined, vortexed for 60 s, and kept at -20°C overnight. Following centrifugation (14,000 ×*g*, 4°C) for 15 min, 30 μl of supernatants was evaporated to dryness under nitrogen stream. The residue was reconstituted in 40 μl of 20 mg/ml methoxyamine hydrochloride in pyridine, and the resulting mixture was incubated at 37°C for 90 min. Next, 40 μl of BSTFA (with 1% TMCS) was added into the mixture and derivatized at 70°C for 60 min prior to GC-MS metabolomics analysis. Aliquots of quality control (QC) sample pooled from all samples were prepared and analyzed with the same procedure as those of the experiment samples.

### GC-MS Analysis

Metabolomics instrumental analysis was performed on an Agilent 7890A gas chromatography system coupled to an Agilent 5975C inert MSD system (Agilent Technologies Inc.). An OPTIMA 5 MS Accent fused-silica capillary column (30 m × 0.25 mm × 0.25 μm; MACHEREY-NAGEL, Germany) was utilized to separate the derivatives. Helium (>99.999%) was used as a carrier gas at a constant flow rate of 1 ml/min through the column. Injection volume was 1 μl, and the solvent delay time was 6 min. The initial oven temperature was held at 70°C for 2 min, ramped to 160°C at a rate of 6°C/min, to 240°C at a rate of 10°C/min, to 300°C at a rate of 20°C/min, and finally held at 300°C for 6 min. The temperatures of injector, transfer line, and electron impact ion source were set to 250°C, 260°C, and 230°C, respectively. The electron ionization (EI) energy was 70 eV, and data were collected in a full-scan mode (m/z 50-600).

### ACE-Inhibitory Activity

The probiotic fermented milk samples made with *L. plantarum* K25 were centrifuged (10,000 ×*g*, 20 min, 4°C) to obtain supernatants containing peptide fractions [25]. After adjusting the supernatant pH to 7.2 and diluting the supernatant 100 times with PBS (pH = 7.2), the ACE-inhibitory activity of the supernatants was determined spectrophotometrically as reported by Ronca-Testoni (1983) using the tripeptide, 2-furanacryloylephenylalanylglycylglycine (FAPGG) as substrate, with some modifications [26]. Briefly, 100 μl of FAPGG solution (1.66 mmol/l in reaction buffer containing 100 mmol/l Tris HCl, 0.6 mol/l NaCl, pH 7.2), was mixed directly in a 96-well microplate with 85 μl of the same reaction buffer or 85 μL of samples. The solution was kept at 37°C for 3 min before adding 15 μL of ACE solution. The reaction was monitored at 345 nm for 10 min. The ACE inhibitory activity was calculated as percent of inhibition (ACEi%). The ACEi activity was calculated as follows:

$$\mathrm{ACEi}(\%)=(1-\frac{{\mathrm{\Delta A}}_{\mathrm{sample}}}{{\mathrm{\Delta A}}_{\mathrm{control}}})\times 100$$

where A_*control*_ is the absorbance of negative control (without samples), A_*sample*_ is the absorbance of the probiotic sample.

### Data Analysis

Triplicate trials were carried out with each sample of 5 replicates. The peak picking, alignment, deconvolution, and further processing of raw GC-MS data were conducted by referring to previous published protocols [27]. The normalized data analysis was performed using SIMCA software (version 14.1, Umetrics, Sweden). For univariate statistical analysis, the normalized data were calculated by Student’s *t*-test in EXCEL. The AMDIS software was applied to deconvolute mass spectra from raw GC-MS data. Two-way analysis of variance (ANOVA) followed by Tukey’s post hoc test was used to determine significant differences (*p* < 0.05) among means. All analyses were performed with GraphPad Prism version 6.00 (GraphPad software, USA). The AMDIS software was applied to deconvolute mass spectra from raw GC-MS data, and the purified mass spectra were automatically matched with an in-house standard library including retention time and mass spectra, Golm Metabolome Database, and Agilent Fiehn GC/MS Metabolomics RTL Library.

## Results and Discussion

### Probiotic Survivability and pH Change in Fermented Milk

**Fig. 1:**
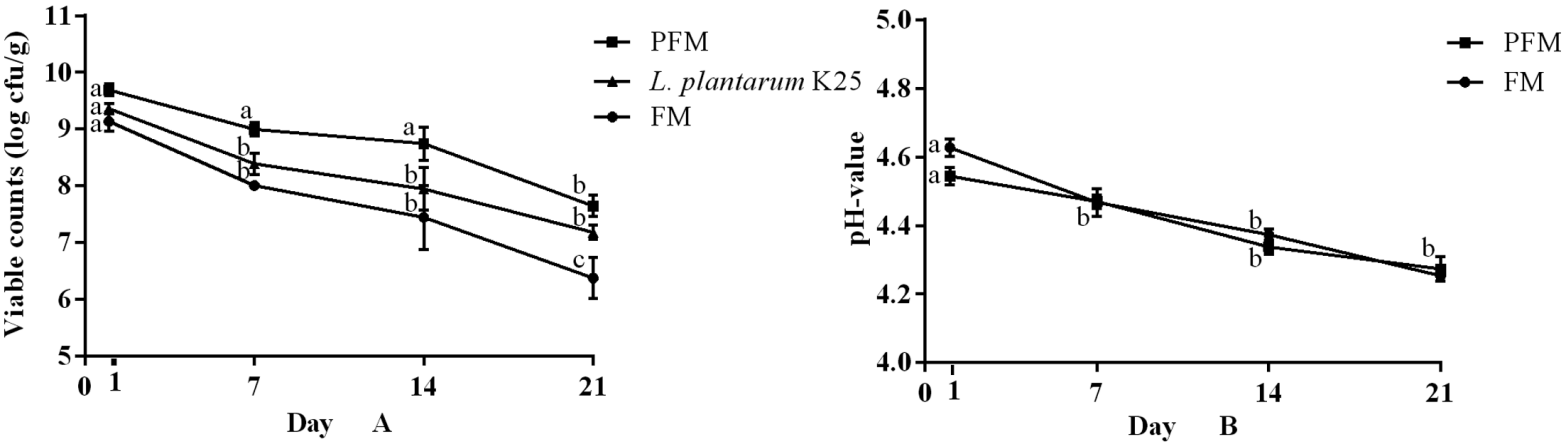
Changes in viable count (A) and pH (B) of FM (fermented milk made with yogurt starter) and PFM (fermented milk made with yogurt starter and probiotic *L. plantarum* K25) during 21 days of storage at 4°C.

Change of bacterial viable counts in the samples of FM and PFM throughout the 21 days of storage at 4°C was shown in Fig. 1A. At the first day of storage, the viable counts of both the FM and PFM samples were above 9 log CFU/g with higher count in the latter. Gradual decrease in the viable counts was observed in all the samples, but the PFM sample maintained significantly higher counts than the FM sample during the whole period of storage. At the end of storage (day 21), the FM and PFM samples had viable counts of 6.4 ± 0.4 log CFU/g and 7.6 ± 0.2 log CFU/g, respectively, while the viable count of *L. plantarum* K25 in the PFM sample was 7.1 ± 0.1 log CFU/g, suggesting good survivability of the probiotic strain in fermented milk. Other probiotic strains were also shown to survive well in fermented milk. For example, in yogurt made with co-culture of probiotic *Bifidobacterium animalis* subsp. *lactis* BB12 or *L. rhamnosus* GG, the viable counts in both the probiotic fermented milk samples remained stable (above 8.0 log CFU/g) throughout 21 days of refrigerated storage [23]. However, probiotic strains *L. delbrueckii* ssp. *bulgaricus* 1932 and *L. rhamnosus* PRA331 were found to decrease from 8.52 ± 0.16 log CFU/g to 6.50 log CFU/g, and from 9.08 ± 0.09 log CFU/g to 7.29 ± 0.13 log CFU/g, respectively, during the 21 days of refrigerated storage [25].

Changes in pH of the fermented milk samples were monitored during the storage (Fig. 1B). Similar pH decrease patterns were observed in both the FM and PFM samples with the pH values of 4.3 and 4.2, respectively, at the end of storage. This suggested that addition of probiotic *L. plantarum* K25 did not cause post-acidification of fermented milk during the refrigerated storage. Previously, several probiotic strains such as *L. plantarum* WCFS1, *L. rhamnosus* GG and *Bifidobacterium animalis* subsp. *lactis* BB12 demonstrated a similar pattern of pH decrease in fermented milk during fermentation with a final pH value of about 4.1 after 21 days of refrigerated storage [28].

### Volatile Metabolite Profiles Determined by Headspace SPME-GC/MS

Change of volatile metabolite profiles in the FM and PFM samples during 21 days of refrigerated storage was shown in Table 1. Among various volatile compounds detected, acetoin and 2,3-butanediol, which are known as C4 compounds responsible for the typical aroma of fermented milk, were found at relatively high concentrations during the storage. These compounds could be generated from glycolysis or citrate metabolism in several lactic acid bacteria such as *Lactococcus*, *Leuconostoc*, and *Weissella* species. Acetoin was significant for reducing the harshness of diacetyl and contributed to the mild creamy flavor [29]. 2,3-Butanediol was the reduced form of acetoin that had limited contribution to the creamy or buttery attribute [30].

Table 1 also shows that there were many other volatile compounds detected in the probiotic fermented milk including aldehydes, acids, alcohols and phenols. Among these volatiles, aldehydes (butanal, heptanal octanal, nonanal, decanal, benzaldehyde), alcohols (3-hexanol, hexanol, octanol, benzyl alcohol) and phenols that might be derived from free amino acids produced by proteolysis could contribute to yogurt flavor [30]. Previous studies showed that probiotic *L. rhamnosus* GG did not significantly influence the major aroma-forming volatile metabolites in fermented milk, but it contributed to formation of volatile and non-volatile organic acids, and free amino acids [32]. Fermented milk with *L. casei* had predominant volatiles such as acetic acid, butyric acid, caproic acid, 2-pentanone, and 2-butanone, while the volatile compounds typical of yogurt were absent [33].

### Analysis of Differential Metabolites before Storage of Fermented Milk

**Fig. 2:**
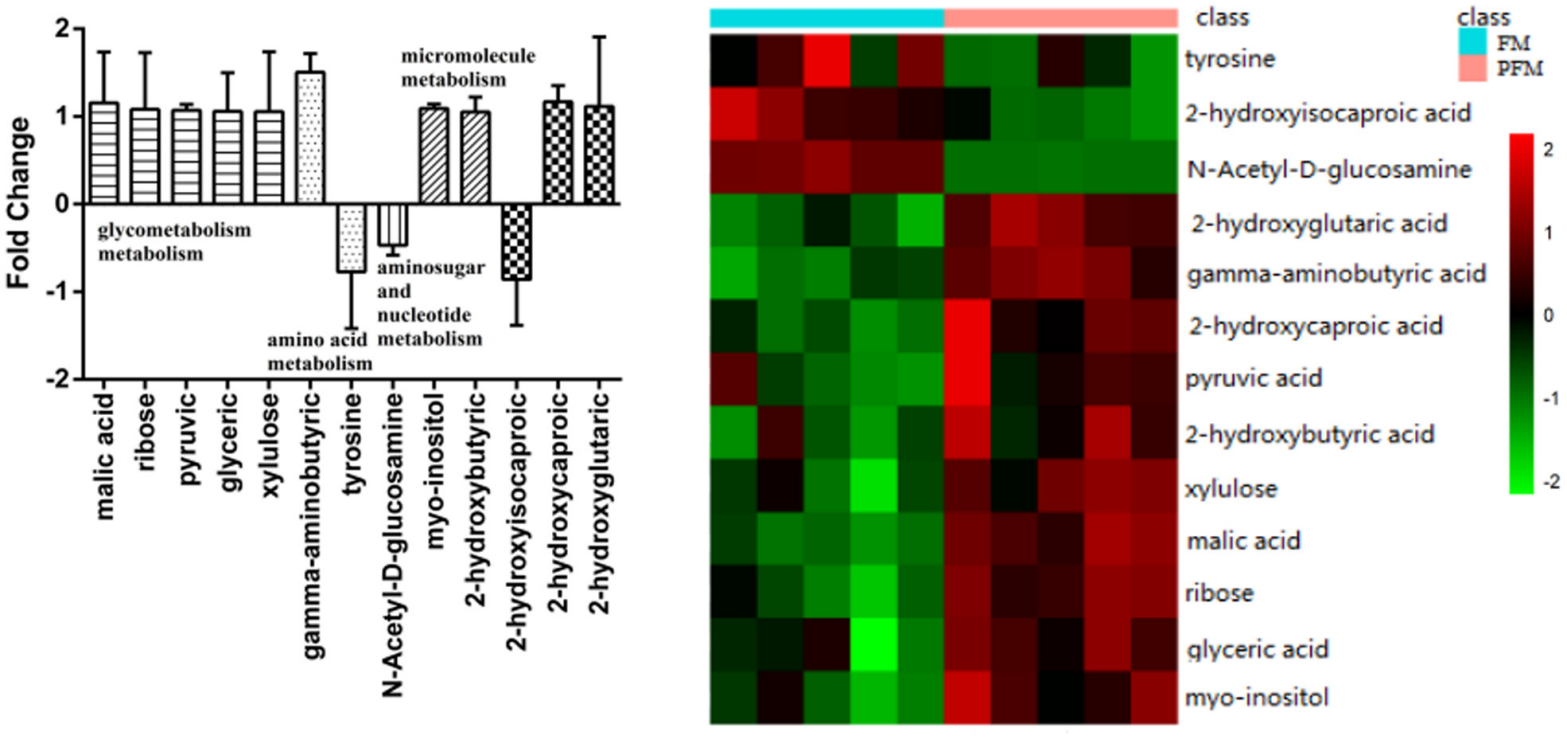
Difference in metabolites between the FM (fermented milk made with yogurt starter) and PFM (fermented milk made with yogurt starter and probiotic *L. plantarum* K25) samples before storage. The positive axis indicates increased production of the metabolites in the PFM sample with *L. plantarum* K25, and the negative axis indicates increased production of the metabolites in the FM samples.

Difference in metabolic products between the samples of FM and PFM with *L. plantarum* K25 before storage was analyzed by metabolomics method (Fig. 2). Considerable difference was observed between the 2 samples with significantly higher levels in the PFM sample of GABA, 2-hydroxycaproic acid, malic acid, 2-hydroxyglutaric acid, myo-inositol, ribose, pyruvic acid, glyceric acid, xylulose, and 2-hydroxybutyric acid. High levels of 2-hydroxyisocaproic acid, tyrosine and *N*-acetyl-D-glucosamine were found in the FM sample. These might involve several metabolic pathways, such as glycometabolism, amino acid metabolism, aminosugar and nucleotide metabolisms, and other micromolecule metabolisms. Among these metabolites, the highest level of GABA in the PFM sample was observed, indicating that *L. plantarum* K25 played the main role in formation of GABA. There were many reports on GABA-rich foods such as cheese, yogurt, soy milk, and fermented bread [34-36]. GABA had physiological functions in important processes such as neurotransmission and antihypertensive activities, thus decreasing anxiety, fear and depression, as well as delaying or inhibiting cancer cell invasion, improving memory, and healing cutaneous wounds [20, 37]. Many lactic acid bacterial strains, such as *L. brevis* DPC6108, *L. brevis* PM17, *L. plantarum* C48, *L. paracasei* PF6 and *L. lactis* PU1, were shown to synthesize GABA when they were grown in culture medium supplemented with monosodium glutamate [35, 38].

### Analysis of Metabolite Changes during Storage of Fermented Milk

To further understand the mechanism of formation of metabolites by probiotic *L. plantarum* K25 in fermented milk, the metabolic changes during 21 days of refrigerated storage were further studied by metabolomics analysis of the PFM samples. Table 2 shows change of the metabolites in the PFM samples during the storage.

**Fig. 4:**
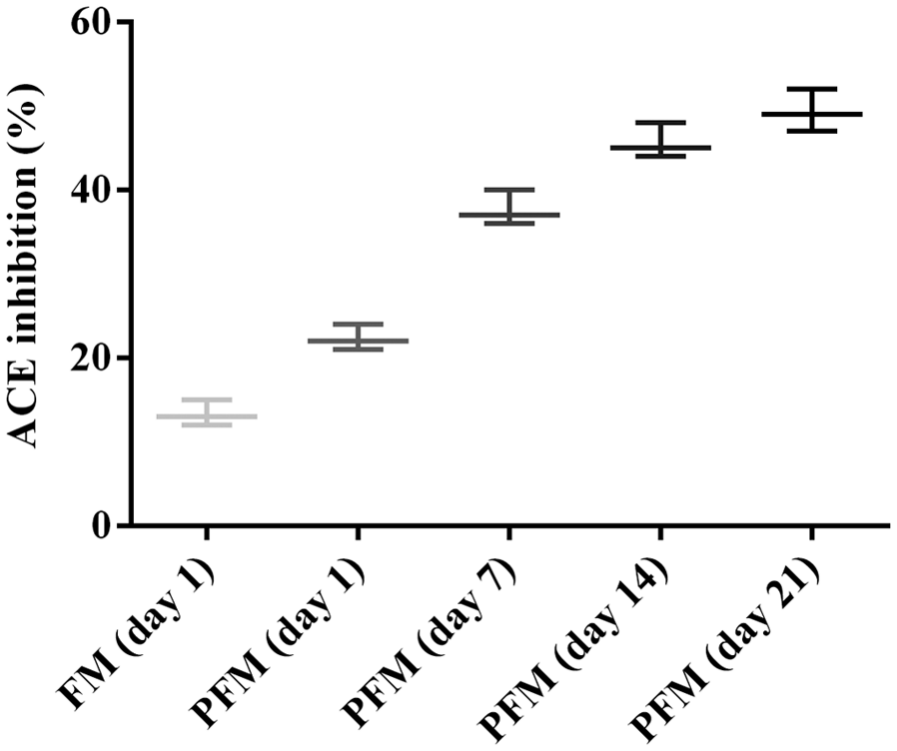
ACE inhibition activity of the FM (fermented milk made with yogurt starter) and PFM (fermented milk made with yogurt starter and probiotic *L. plantarum* K25) samples during 21 days of storage at 4°C.

Among the metabolites detected, the major sugars were 1,3-dihydroxyacetone, ribose, and xylulose in the PFM sample. The concentrations of 1,3-dihydroxyacetone and xylulose decreased during the storage probably due to bacterial utilization of sugars for energy to survive. Ribose, mainly formed from hydrolysis of RNA, might not be used by the microbes, as reported earlier with *L. amylovorus* NCFB 2745, which could not utilize ribose [39]. Furthermore, increases in amino acids (GABA, glycine, alanine, ornithine, tyrosine, 2-ketoglutaric acid and fumaric acid), and organic acids (malic acid, 2-hydroxyisocaproic acid, 2-hydroxyisovaleric acid, glyceric acid, succinic acid and citric acid) were also observed during the whole storage period. Although many metabolites were directly produced by degradation of proteins and other large molecules independent of the proposed pathway, the major functional metabolite GABA was produced by *L. plantarum* K25 involving the amino acid metabolic pathway. In addition, some metabolites employed in storage might use the metabolic pathway to acquire energy, carbon, and nitrogen sources. On the basis of the metabolites found in the present study, the relevant metabolic pathways in *L. plantarum* K25 were constructed (Fig. 4), including the amino acid degradation pathway, citrate cycle (TCA cycle), pentose phosphate pathway (PPP) and urea cycle.

Organic acids, amino acids and sugars are known to be directly associated with taste and flavor quality, as well as the functional properties of fermented products. The increased formation of these metabolites as observed in this study might modify the sensory quality and functionality of the probiotic *L. plantarum* K25 fermented milk. Previously, increased formation of several organic acids in fermented foods during storage were also detected by GC/MS [40-41]. Glyceric acid was a metabolite from a product of fructose breakdown, and it had liver stimulant and cholesterolytic activity [42]. Malic acid was reported with many physiological functions such as antioxidant activity to capture free radicals, and promoting the absorption of anti-cancer drugs and calcium [43]. Succinic acid could inhibit passive and active skin allergic reactions and reduce the formation of IgE antibodies in animal serum [44]. Significant increase in formation of amino acids, such as alanine, leucine, glycine and tyrosine, was also reported in fermented milk with probiotic *L. plantarum* WCFS1 during storage [45]. It was of interest that the content of glycine increased to a relatively high level in the PFM sample with *L. plantarum* K25, but this was not reported in other probiotic fermented milk. Glycine was reported to be the basic structure of many important substances such as methionine, serine, threonine, vitamin B6, and deoxyribonucleic acid, and it had the function of preventing Parkinson’s disease, chronic enteritis, and gastric acid overload, as well as treating cardiovascular diseases [46-50]. Glycine-alanine complexes were shown with antitumor activity [51]. Alanine could prevent kidney stones, assist glucose metabolism, help ease hypoglycemia, and improve physical energy [52]. Furthermore, an increased level of ribose in the probiotic *L. plantarum* K25 fermented milk might be beneficial since oral administration of ribose was reported to improve athletic performance by boosting muscle energy and making it readily available, and also to improve symptoms of diseases such as chronic fatigue syndrome, fibromyalgia and coronary artery disease [53]. Finally, 1,3-dihydroxyacetone was a three-carbon compound in glycolysis that promoted athletic performance [54].

### Angiotensin-Converting Enzyme Inhibitory Activity

Probiotic *L. plantarum* K25 might promote proteolysis in fermented milk as indicated by increased formation of amino acids (Table 2). The degree of proteolysis of fermented milk produced by *Lactobacillus* strains was shown to correlate with the ACEi activity [55]. Good correlation between the degree of hydrolysis and ACEi activity in fermented milk by *L. casei* YIT 9029 was also reported [56]. In this study, bioactivity of the fermented milk with *L. plantarum* K25 was further assayed by determining ACEi activity that was often related to anti-hypertensive hydrolysates of milk proteins [57]. As shown in Fig. 4, the ACEi activity of the PFM sample with *L. plantarum* K25 was significantly higher than that of the FM sample. Gradual increase in the ACEi activity from 22.3% to 49.3% was observed in the PFM sample during 21 days of storage, suggesting formation of bioactive metabolites in the fermented milk during the storage. Therefore, correlation between the metabolites and ACEi activity of the PFM sample during the storage was further analyzed. As shown in Table 2, several metabolites showed relatively strong positive correlation (coefficient > 0.70) with the ACEi activity, such as GABA, glyceric acid, malic acid, succinic acid, pyroglutamic acid and leucine. Relatively strong negative correlation (coefficient > - 0.70) to the ACEi activity was found with 2-ketoglutaric acid, fumaric acid, pyruvic acid, ornithine and 1,3-dihydroxyacetone.

Previously, ACEi activity was found to increase gradually during cold storage of probiotic fermented milk [58]. The permeate of probiotic fermented goat milk was also shown to possess high ACEi activity [55]. *L. casei* ATCC 393 showed high ACEi activity in fermented soy milk [59]. In this study, the ACEi activity of the fermented milk with *L. plantarum* K25 was comparable to those reported for other probiotics such as *L. casei* L26 and LC279 [60], and *L. casei* FC113 [61]. GABA derived from L-glutamic acid was shown to possess ACEi activity [62]. However, the roles of other metabolites mentioned above in association with the ACEi activity of the PFM with *L. plantarum* K25 need to be further studied.

## Figures and Tables

**Fig. 3:**
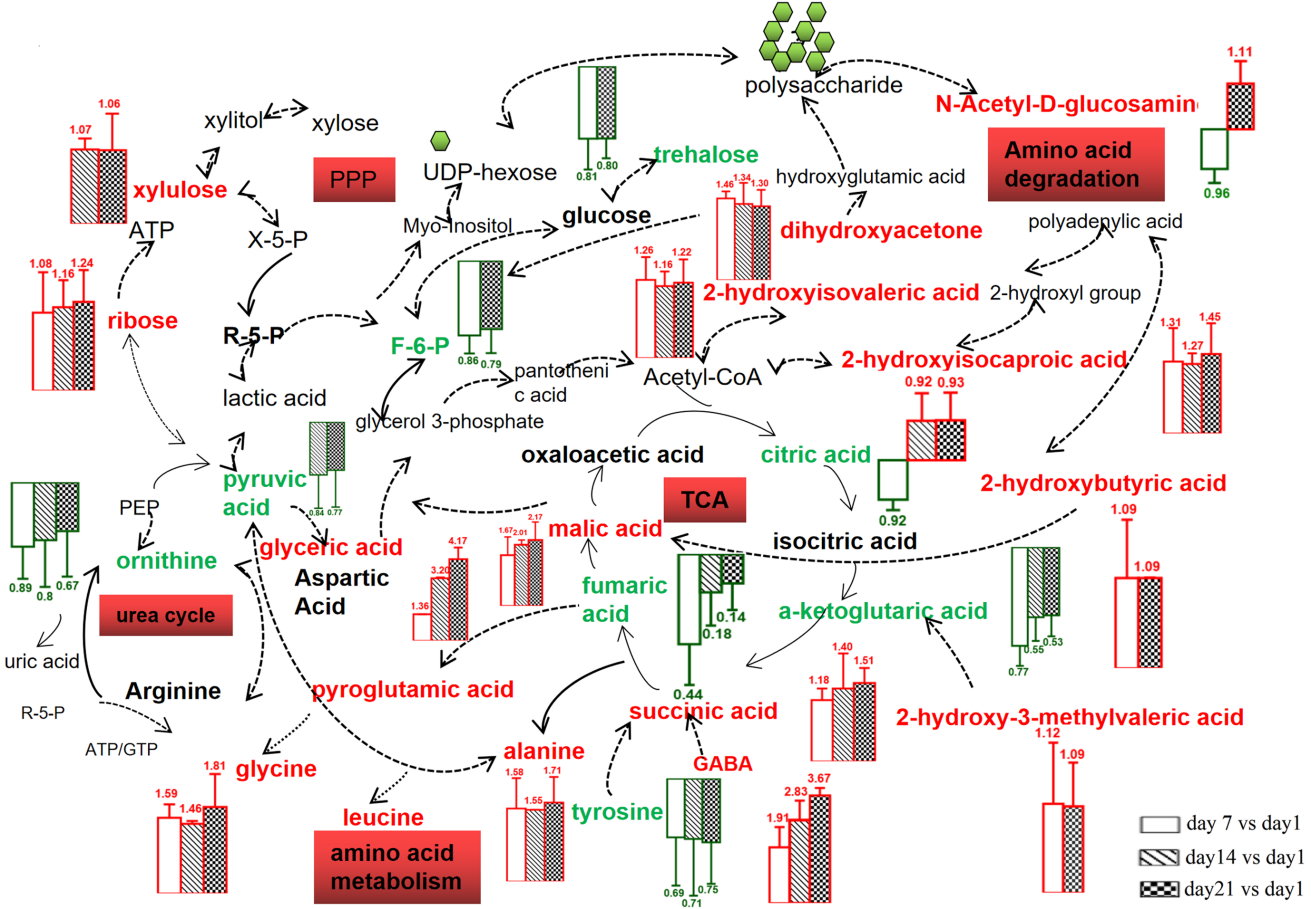
Schematic representation of metabolites produced in PFM (fermented milk made with yogurt starter and probiotic *L. plantarum* K25) sample during 21 days of storage at 4°C. Metabolites identified by UPLS-Q-TOF are marked red and green. Red represents increased metabolites, and green represents decreased metabolites.

**Table 1:** Identification of volatile metabolites by GC-MS and their changes during refrigerated storage of the FM (fermented milk made with yogurt starter) and PFM (fermented milk made with yogurt starter and probiotic *L. plantarum* K25) samples.

Identification	Chemical formula	Content (ng/g)				

		FM	PFM			

		Day1	Day1	Day7	Day14	Day21
Butanal	C_4_H_8_O	0.73 ± 0.01a	−	−	−	−
2,3-Butanedione	C_4_H_6_O_2_	2.32 ± 0.11c	1.95 ± 0.03e	1.72 ± 0.2d	3.29 ± 0.06b	4.42 ± 0.07a
2,3-Pentanedione	C_5_H_8_O_2_	3.50 ± 0.03a	−	−	−	−
2-Heptanone	C_7_H_14_O	0.84 ± 0.04b	0.65 ± 0.01c	1.08 ± 0.09a	0.26 ± 0.02d	0.34 ± 0.11d
Limonene	C_10_H_16_	0.72 ± 0.01a	0.72 ± 0.02a	0.72 ± 0.02a	0.63 ± 0.1a	0.67 ± 0.08a
Styrene	C_8_H_8_	−	−	−	−	0.66 ± 0.04a
Heptanal	C_7_H_14_O	−	0.19 ± 0.01a	0.07 ± 0.02c	0.08 ± 0.01c	0.13 ± 0.04b
Acetoin	C_4_H_8_O_2_	5.12 ± 0.05c	4.16 ± 0.04d	5.87 ± 0.16b	7.71 ± 0.19a	7.53 ± 0.14a
Octanal	C_8_H_16_O	0.84 ± 0.03a	−	−	0.18 ± 0.02b	0.05 ± 0.01c
3-Hexanol	C_6_H_14_O	0.432 ± 0.01c	0.65 ± 0.02a	0.48 ± 0.03b	0.46 ± 0.01bc	0.65 ± 0.04a
Hexanol	C_6_H_14_O	−	0.20 ± 0.01ab	0.14 ± 0.04c	0.16 ± 0.02bc	0.21 ± 0.01a
2-Nonanone	C_9_H_18_O	0.83 ± 0.02c	1.31 ± 0.08a	1.04 ± 0.03b	0.64 ± 0.04d	0.50 ± 0.13e
Nonanal	C_9_H_18_O	2.26 ± 0.18a	0.45 ± 0.01c	0.22 ± 0.01d	1.26 ± 0.19b	0.46 ± 0.08c
Acetic acid	C_2_H_4_O_2_	4.15 ± 0.05e	20.30 ± 0.06c	17.40 ± 0.09d	31.32 ± 0.07a	26.81 ± 0.16b
Heptanol	C_7_H_16_O	0.16 ± 0.05c	0.34 ± 0.01b	0.25 ± 0.03bc	0.22 ± 0.03c	0.68 ± 0.12a
1-Hexanol, 2-ethyl-	C_8_H_18_O	1.86 ± 0.04b	1.19 ± 0.07c	0.91 ± 0.01d	1.23 ± 0.19c	1.59 ± 0.03a
Decanal	C10H20O	1.86 ± 0.06a	1.16 ± 0.03c	0.94 ± 0.04d	1.10 ± 0.08c	1.62 ± 0.01b
Benzaldehyde	C_7_H_6_O	5.58 ± 0.13a	2.53 ± 0.02b	1.48 ± 0.01d	1.08 ± 0.11e	2.30 ± 0.1c
Octanol	C_8_H_18_O	−	0.49 ± 0.02c	0.89 ± 0.09a	0.53 ± 0.03c	0.74 ± 0.04b
2-Undecanone	C11H22O	0.43 ± 0.02c	0.50 ± 0.01b	0.29 ± 0.06d	0.38 ± 0.01c	0.79 ± 0.01a
Butyrolactone	C_4_H_6_O_2_	0.93 ± 0.01a	0.96 ± 0.01a	0.58 ± 0.07c	0.72 ± 0.02b	0.38 ± 0.01d
Ethanol, 2-(2-ethoxyethoxy)-	C6H14O_3_	3.83 ± 0.05a	3.39 ± 0.01b	2.40 ± 0.2d	2.39 ± 0.03d	2.81 ± 0.09c
Butanoic acid	C_4_H_8_O_2_	1.32 ± 0.08a	1.07 ± 0.03c	0.87 ± 0.02d	0.65 ± 0.02e	1.18 ± 0.07b
Acetophenone	C_8_H_8_O	−	0.61 ± 0.01a	0.26 ± 0.03b	−	−
Nonanol	C9H_20_O	0.97 ± 0.03c	0.54 ± 0.02e	0.76 ± 0.02d	1.32 ± 0.17b	1.88 ± 0.04a
Hexanoic acid	C_6_H_12_O_2_	4.88 ± 0.03b	5.39 ± 0.16a	4.22 ± 0.09c	3.75 ± 0.08d	5.25 ± 0.16a
Benzyl alcohol	C_7_H_8_O	0.04 ± 0.04b	0.15 ± 0.01ab	0.07 ± 0.03b	0.34 ± 0.26a	0.07 ± 0.03b
Benzothiazole	C_7_H_5_NS	−	−	−	0.74 ± 0.19b	0.97 ± 0.03a
Heptanoic acid	C_7_H_14_O_2_	0.36 ± 0.01a	0.17 ± 0.01e	0.24 ± 0.01d	0.32 ± 0.03b	0.29 ± 0.02c
Phenol	C_6_H_6_O	0.32 ± 0.01a	0.29 ± 0.02ab	0.26 ± 0.03b	0.23 ± 0.04b	0.24 ± 0.03b
Octanoic acid	C_8_H_16_O_2_	8.39 ± 0.03c	9.57 ± 0.17b	7.13 ± 0.06d	6.83 ± 0.1e	9.99 ± 0.08a
Nonanoic acid	C9H_18_O_2_	1.33 ± 0.01b	0.28 ± 0.02c	0.22 ± 0.02d	1.84 ± 0.12a	1.35 ± 0.11b
n-Decanoic acid	C_10_H_20_O_2_	0.65 ± 0.02d	1.46 ± 0.04c	2.52 ± 0.06b	2.57 ± 0.04b	2.84 ± 0.11a
Benzoic acid	C_7_H_6_O_2_	6.69 ± 0.01b	6.89 ± 0.19a	4.927 ± 0.01c	4.53 ± 0.01d	4.32 ± 0.07e

Values presented are means ± standard deviation. Means in the same row followed by different letters are significantly different (*p* < 0.05).

**Table 2:** Metabolomics analysis of the changes of metabolites and their correlation to ACE-inhibitory activity in the probiotic fermented milk (PFM) with *L. plantarum* K25 during 21 days of refrigerated storage.

Metabolites	PFM			Correlation coefficient

	Fold change			

	Day 7 vs Day 1	Day 14 vs Day 1	Day 21 vs Day 1	
γ-Aminobutyric acid	↑ (0.93c)	↑ (1.50b)	↑ (1.88a)	0.74
Alanine	↑ (0.66b)	↑ (0.63c)	↑ (0.77a)	0.36
Glycine	↑ (0.67c)	↑ (0.55b)	↑ (0.86a)	0.28
Glyceric acid	↑ (0.44c)	↑ (1.68b)	↑ (2.06a)	0.74
Malic acid	↑ (0.74c)	↑ (1.01b)	↑ (1.12a)	0.72
Succinic acid	↑ (0.24c)	↑ (0.49b)	↑ (0.59a)	0.72
2-Hydroxy-3-methylvaleric acid	↑ (0.16a)	——	↑ (0.12b)	-0.41
2-Hydroxybutyric acid	↑ (0.12b)	↑ (0.22a)	——	-0.31
2-Hydroxyisocaproic acid	↑ (0.39b)	↑ (0.34c)	↑ (0.54a)	0.36
2-Hydroxyisovaleric acid	↑ (0.34a)	↑ (0.22c)	↑ (0.29b)	-0.55
Pyroglutamic acid	——	↑ (0.19b)	↑ (0.28a)	0.73
2-Ketoglutaric acid	↓ (0.37c)	↓ (0.87b)	↓ (0.91a)	-0.76
Fumaric acid	↓ (1.21c)	↓ (2.46b)	↓ (2.84a)	-0.76
Citric acid	——	↓ (0.11b)	↑ (0.10a)	0.10
Pyruvic acid	——	↓ (0.25a)	↓ (0.39a)	-0.78
1,3-Dihydroxyacetone	↑ (0.52a)	↑ (0.42b)	↑ (0.38c)	-0.84
Ribose	↑ (0.12c)	↑ (0.21b)	↑ (0.30a)	0.67
*N*-Acetyl-D-glucosamine	↑ (0.06b)	——	↑ (0.15a)	0.26
Xylulose	——	↑ (0.10a)	↑ (0.08b)	0.57
Fructose-6-phosphate	↓ (0.21b)	——	↓ (0.34a)	-0.16
Trehalose	↓ (0.31a)	——	↓ (0.31a)	0.13
Glycerol	↓ (0.19b)	——	↓ (0.22a)	0.03
Ethanolamine	↓ (0.24a)	↓ (0.15b)	↓ (0.23b)	0.10
Leucine	——	↑ (0.26b)	↑ (0.38a)	0.73
Ornithine	↓ (0.17c)	↓ (0.33b)	↓ (0.57a)	-0.75
Tyrosine	↓ (0.54a)	↓ (0.50b)	↓ (0.42c)	0.59

Data in the same row followed by different letters are significantly different (*p* < 0.05).

## References

[1] Laws G, Kemp R (2019). Probiotics and health: understanding probiotic trials. N. Z. Med. J..

[2] Nicholson JK, Lindon JC, Holmes E (1999). Metabonomics': understanding the metabolic responses of living systems to pathophysiological stimuli via multivariate statistical analysis of biological NMR spectroscopic data. Xenobiotica.

[3] Niwa T (1986). Metabolic profiling with gas chromatography-mass spectrometry and its application to clinical medicine. J. Chromatogr..

[4] Schoina V, Terpou A, Angelika-Ioanna G, Koutinas A, Kanellaki M, Bosnea L (2015). Use of *Pistacia terebinthus* resin as immobilization support for *Lactobacillus casei* cells and application in selected dairy products. J. Food Sci. Technol..

[5] Jewett MC, Hofmann G, Nielsen J (2006). Fungal metabolite analysis in genomics and phenomics. Curr. Opin. Biotechnol..

[6] Kieserling K, Vu TM, Drusch S, Schalow S (2019). Impact of pectin-rich orange fibre on gel characteristics and sensory properties in lactic acid fermented yoghurt. Food Hydrocoll..

[7] Pradhan D, Mallappa RH, Grover S (2020). Comprehensive approaches for assessing the safety of probiotic bacteria. Food Control.

[8] Wishart DS (2008). Metabolomics: applications to food science and nutrition research. Trends Food Sci. Technol..

[9] Lindon JC (2007). The Handbook of Metabonomics and Metabolomics.

[10] Madsen K (2011). Using metabolomics to decipher probiotic effects in patients with irritable bowel syndrome. J. Clin. Gastroenterol..

[11] Hong Y, Hong KS, Park M, Ahn Y, Lee J, Huh C (2011). Metabonomic understanding of probiotic effects in humans with irritable bowel syndrome. J. Clin. Gastroenterol..

[12] Zhang L, Zhang X, Liu C, Li C, Li S, Li T (2013). Manufacture of Cheddar cheese using probiotic *Lactobacillus plantarum* K25 and its cholesterol-lowering effects in a mice model. World J. Microbiol. Biotechnol..

[13] Wang J (2012). Tenchnological properties of *Lactobacillus plantarum* K25. J. Food Sci. Biotechnol..

[14] Zhao YL (2012). Acuteoral toxicity and bacterial translocation evaluation of *Lactobacillus plantarum* K25. Dairy Ind. China.

[15] Jiang Y, Zhang J, Zhao X, Zhao W, Yu Z, Chen C (2018). Complete genome sequencing of exopolysaccharide-producing *Lactobacillus plantarum* K25 provides genetic evidence for the probiotic functionality and cold endurance capacity of the strain. Biosci. Biotechnol. Biochem..

[16] Gonzalez-Gonzalez C, Gibson T, Jauregi P (2013). Novel probiotic-fermented milk with angiotensin I-converting enzyme inhibitory peptides produced by *Bifidobacterium bifidum* MF 20/5. Int. J. Food Microbiol..

[17] Nejati F, Rizzello CG, Di CR, Sheikh-Zeinoddin M, Diviccaro A, Minervini F (2013). Manufacture of a functional fermented milk enriched of Angiotensin-I Converting Enzyme (ACE)-inhibitory peptides and gamma-amino butyric acid (GABA). LWT-Food Sci. Technol..

[18] Moslehishad M, Ehsani MR, Salami M, Mirdamadi S, Ezzatpanah H, Naslaji AN (2013). The comparative assessment of ACEinhibitory and antioxidant activities of peptide fractions obtained from fermented camel and bovine milk by *Lactobacillus rhamnosus* PTCC 1637. Int. Dairy J..

[19] Meira SMM, Daroit DJ, Helfer VE, Correa APF, Segalin J, Carro S (2012). Bioactive peptides in water-soluble extracts of ovine cheeses from Southern Brazil and Uruguay. Food Res. Int..

[20] Inoue K, Shirai T, Ochiai H, Kasao M, Hayakawa K, Kimura M (2003). Blood-pressure-lowering effect of a novel fermented milk containing gamma-aminobutyric acid (GABA) in mild hypertensives. Eur. J. Clin. Nutr..

[21] Taubert D, Roesen R, Schoemig E (2007). Effect of cocoa and tea intake on blood pressure-A meta-analysis. Arch. Int. Med..

[22] Bujalance C, Jiménez-Valera M, Moreno E, Ruiz-Bravo A (2006). A selective differential medium for *Lactobacillus plantarum*. J. Microbiol. Meth..

[23] Settachaimongkon S, Nout MJR, Fernandes ECA, Hettinga KA, Vervoort JM, Zwietering MH (2014). Influence of different proteolytic strains of *Streptococcus thermophilus* in co-culture with *Lactobacillus delbrueckii* subsp. bulgaricus on the metabolite profile of set-yoghurt. Int. J. Food Microbiol..

[24] Leksrisompong P, Barbano DM, Foegeding AE, Gerard P, Drake M (2010). The roles of fat and pH on the detection thresholds and partition coefficients of three compounds: diacetyl, delta-decalactone and furaneol. J. Sens. Stud..

[25] Rutella GS, Tagliazucchi D, Solieri L (2016). Survival and bioactivities of selected probiotic lactobacilli in yogurt fermentation and cold storage: New insights for developing a bi-functional dairy food. Food Microbiol..

[26] Ronca-Testoni S (1983). Direct spectrophotometric assay for angiotensin-converting enzyme in serum. Clin. Chem..

[27] Gao X, Pujos-Guillot E, Sebedio J (2010). Development of a quantitative metabolomic approach to study clinical human fecal water metabolome based on Trimethylsilylationd derivatization and GC/MS analysis. Anal. Chem..

[28] Settachaimongkon S, Winata V, Wang X, Nout MJR, Zwietering MH, Smid EJ (2015). Effect of sublethal preculturing on the survival of probiotics and metabolite formation in set-yoghurt. Food Microbiol..

[29] Hefa C (2010). Volatile flavor compounds in yogurt: a review. Crit. Rev. Food Sci..

[30] Hugenholtz, J (1993). Citrate metabolism in lactic acid bacteria. FEMS Microbiol. Rev..

[31] Liu M, Bayjanov JR, Renckens B, Nauta A, Siezen RJ (2010). The proteolytic system of lactic acid bacteria revisited: a genomic comparison. BMC Genomics.

[32] Murgia A, Scano P, Cacciabue R, Dessì D, Caboni P (2019). GC-MS metabolomics comparison of yoghurts from sheep's and goats' milk. Int. Dairy J..

[33] Zareba D, Ziarno M, Scibisz I, Gawron J (2014). The importance of volatile compound profile in the assessment of fermentation conducted by *Lactobacillus casei* DN-114 001. Int. Dairy J..

[34] Nomura M, Kimoto H, Someya Y, Furukawa S, Suzuki I (1998). Production of gamma-aminobutyric acid by cheese starters during cheese ripening. J. Dairy Sci..

[35] Siragusa S, De Angelis M, Di Cagno R, Rizzello CG, Coda R, Gobbetti M (2007). Synthesis of gamma-aminobutyric acid by lactic acid bacteria isolated from a variety of Italian cheeses. Appl. Environ. Microbiol..

[36] Li H, Cao Y (2010). Lactic acid bacterial cell factories for gamma-aminobutyric acid. Amino Acids.

[37] Owens DF, Kriegstein AR (2002). Is there more to GABA than synaptic inhibition? Nat. Rev. Neurosci..

[38] Barrett E (2014). This article corrects: gamma-Aminobutyric acid production by culturable bacteria from the human intestine. J. Appl. Microbiol..

[39] Whitley K, Marshall VM (1999). Heterofermentative metabolism of glucose and ribose and utilisation of citrate by the smooth biotype of *Lactobacillus amylovorus* NCFB 2745. Antonie Van Leeuwenhoek..

[40] Namgung HJ, Park HJ, Cho IH, Choi HK, Kwon DY, Shim SM (2010). Metabolite profiling of doenjang, fermented soybean paste, during fermentation. J. Sci. Food Agr..

[41] Lee DE, Lee S, Jang ES, Shin HW, Moon BS, Lee CH (2016). Metabolomic profiles of *Aspergillus oryzae* and *Bacillus amyloliquefaciens* during rice koji fermentation. Molecules.

[42] Handa SS (1986). Natural products and plants as liver protecting drugs. Fitoterapia.

[43] Wu J, Wu Q, Zhang J, Zhang W (2015). New studies and progress of biological function of L-malate. Food Ind..

[44] Duan YF, Wang Y, Zhang JS, Song YX, Wang J (2018). Dietary effects of succinic acid on the growth, digestive enzymes, immune response and resistance to ammonia stress of *Litopenaeus vannamei*. Fish Shellfish Immun..

[45] Settachaimongkon S, van Valenberg HJF, Gazi I, Nout MJR, van Hooijdonk TCM, Zwietering MH (2016). Influence of *Lactobacillus plantarum* WCFS1 on post-acidification, metabolite formation and survival of starter bacteria in set-yoghurt. Food Microbiol..

[46] Maity P, Biswas K, Chattopadhyay I, Banerjee RK, Bandyopadhyay U (2009). The Use of Neem for Controlling Gastric Hyperacidity and Ulcer. Phytother. Res..

[47] Effenberger-Neidnicht K, Jaegers J, Verhaegh R, de Groot H (2014). Glycine selectively reduces intestinal injury during endotoxemia. J. Surg. Res..

[48] Cieslik KA, Sekhar RV, Granillo A, Reddy A, Medrano G, Heredia CP (2018). Improved cardiovascular function in old mice after *N*-acetyl cysteine and glycine supplemented diet: inflammation and mitochondrial factors. J. Gerontol. A-Biol..

[49] Cioffi CL, Guzzo PR (2016). Inhibitors of Glycine Transporter-1: Potential Therapeutics for the Treatment of CNS Disorders. Curr. Top. Med. Chem..

[50] Ospina-Rojas IC, Murakami AE, Oliveira CAL, Guerra AFQG (2013). Supplemental glycine and threonine effects on performance, intestinal mucosa development, and nutrient utilization of growing broiler chickens. Poultry Sci..

[51] Mansouri-Torshizi H, Zareian-Jahromi S, Abdi K, Saeidifar M (2019). Nonionic but water soluble, [Glycine-Pd-Alanine] and [Glycine-Pd-Valine] complexes. Their synthesis, characterization, antitumor activities and rich DNA/HSA interaction studies. J. Biomol. Struct. Dyn..

[52] Nesovic-Ostojic J, Kovacevic S, Spasic S, Lopicic S, Todorovic J, Dincic M (2019). Modulation of luminal L-alanine transport in proximal tubular cells of frog kidney induced by low micromolar Cd^2+^ concentration. Comp. Biochem. Physiol. C Toxicol. Pharmacol..

[53] Fan Y, Yan G, Liu F, Rong J, Ma W, Yang D, Yu Y (2019). Potential role of poly (ADP-ribose) polymerase in delayed cerebral vasospasm following subarachnoid hemorrhage in rats. Exp. Ther. Med..

[54] Ślepokura K, Lis T (2010). Dihydroxyacetone phosphate, DHAP, in the crystalline state: monomeric and dimeric forms. Carbohydr. Res..

[55] Moreno-Montoro M, Olalla-Herrera M, Angel Rufian-Henares J, Gimenez Martinez R, Miralles B, Bergillos T (2017). Antioxidant, ACE-inhibitory and antimicrobial activity of fermented goat milk: activity and physicochemical property relationship of the peptide components. Food Funct..

[56] Gonzalez-Gonzalez CR, Tuohy KM, Jauregi P (2011). Production of angiotensin-I-converting enzyme (ACE) inhibitory activity in milk fermented with probiotic strains: effects of calcium, pH and peptides on the ACE-inhibitory activity. Int. Dairy J..

[57] Amorim FG, Coitinho LB, Dias AT, Friques AGF, Monteiro BL, Rezende LCDD (2019). Identification of new bioactive peptides from Kefir milk through proteopeptidomics: Bioprospection of antihypertensive molecules. Food Chem..

[58] Abdel-Hamid M, Romeih E, Gamba RR, Nagai E, Suzuki T, Koyanagi T (2019). The biological activity of fermented milk produced by *Lactobacillus casei* ATCC 393 during cold storage. Int. Dairy J..

[59] Yeo S, Liong M (2010). Angiotensin I-converting enzyme inhibitory activity and bioconversion of isoflavones by probiotics in soymilk supplemented with prebiotics. Int. J. Food Sci. Nutr..

[60] Donkor OHAV (2007). Proteolytic activity of dairy lactic acid bacteria and probiotics as determinant of viability and in vitro angiotensin-converting enzyme inhibitory activity in fermented milk. Le. Lait..

[61] Nejati F, Rizzello CG, Di CR, Sheikh-Zeinoddin M, Diviccaro A, Minervini F (2013). Manufacture of a functional fermented milk enriched of Angiotensin-I Converting Enzyme (ACE)-inhibitory peptides and gamma-amino butyric acid (GABA). LWT-Food Sci. Technol..

[62] Hagi T, Kobayashi M, Nomura M (2016). Metabolome analysis of milk fermented by γ-aminobutyric acid-producing *Lactococcus lactis*. J. Dairy Sci..

